# Editorial: Insights Into New Strategies to Combat Biofilms

**DOI:** 10.3389/fmicb.2021.742647

**Published:** 2021-09-23

**Authors:** Sujogya Kumar Panda, Silvia Buroni, Vishvanath Tiwari, Luis Cláudio Nascimento da Silva

**Affiliations:** ^1^Center of Environment Climate Change and Public Health, RUSA 2.0, Utkal University, Bhubaneswar, India; ^2^Department of Biology and Biotechnology, University of Pavia, Pavia, Italy; ^3^Department of Biochemistry, Central University of Rajasthan, Ajmer, India; ^4^Laboratory of Microbial Pathogenesis, Universidade Ceuma, São Luís, Brazil

**Keywords:** antimicrobials, biofilm, drug discovery, EPS matrix, ESKAPE, peptides, quorum sensing

Biofilms form a complex layer with defined structures, that attach on biotic or abiotic surfaces, are tough to eradicate and tend to cause some resistance against most antibiotics (Sahoo et al., 2021). Although they occur on all kinds of surfaces in the outside environment, biofilms become problematics due to their ability to colonize the human body (60–80% of all infections are due to biofilm). Several studies confirmed that biofilm-producing bacteria are typically much less sensitive to antimicrobials than planktonic cells (Sahoo et al., 2021; Van Puyvelde et al., 2021). The rapid rising of antimicrobial resistance, and the need for new approaches to fill the gap in antimicrobial drug discovery are alarming (Jouneghani et al., 2020) due to the scarcity of effective drugs to combat the formation of biofilm infections and the need for drug discovery efforts to overcome this challenge (Kipanga et al., 2020). One of the strategies is to increase multi-targeted or combinatorial therapies to fight the multifactorial nature of biofilm development. Among bacteria, several pathogenic species are highlighted by their biofilm potential, such as those belonging to the genus *Acinetobacter, Pseudomonas, Vibrio, Listeria, Staphylococcus, Streptococcus, Streptomyces*, etc. Similarly, fungi, such as *Candida* and *Cryptococcus*, are also capable of forming biofilms. The focus of this Research Topic is to make a special contribution to the discovery of novel compounds to combat biofilm both for bacteria and fungi. This special issue consists of 27 articles covering diverse topics.

## Anti-Staphylococcal and Anti-Biofilm Compounds

*Staphylococcus aureus* is a very common pathogen and the evolution of antibiotic resistance in this bacterium has shown that there is no long-lasting remedy especially for hospital-acquired infections. A wound infection by methicillin-resistant *S. aureus* (MRSA) may lead to sepsis and even to death (Panda et al., 2020).

Biofilm-associated infections are one of the major concerns for the healthcare system. Most of the current therapies available are mainly focused on the biocidal approach. Pinto et al. highlighted the strategies to target and disrupt extracellular polymeric substances (EPS). They suggested to use matrix disruptive agents, nanocarriers, magnetic fields, photodynamic therapy, and ultrasounds to control the EPS of biofilms. They propose that a synergistic approach between antibiotics and EPS disruptors can completely eradicate biofilms.

Rembe et al. studied the efficacy of three hypochlorous irrigation solutions against bacteria such as *S. aureus*, Methicillin-Resistant *S. aureus* (MRSA) and *Pseudomonas aeruginosa* using an advanced complex *in-vitro* human plasma biofilm model (hpBIOM). With the help of scanning electron microscopy (SEM), these authors concluded that both reference agents (polyhexanide and octenidine dihydrochloride/phenoxyethanol) induced complete eradication of *P. aeruginosa* and MRSA biofilms after 72 h compared to other tested hypochlorous wound irrigation solutions. The complex hpBIOM model mimics the highly challenging clinical wound micro-environment, providing a profound base for future clinical translation (Rembe et al.).

In a very elegant research, Gangwar et al. studied for the first time the activity of glabridin (C_20_H_20_O_4_, an isoflavane commonly present on *Glycyrrhiza glabra* root) against biofilm formation of MRSA strains. Crystal violet assay and SEM results suggested that glabridin prevents the formation of cell clusters and the attachment of MRSA to the surface in a dose dependent manner. Further studies on proteomic analysis of biofilm matrix by LC-ESI-QTOF confirmed the involvement of several proteins in cells adhesion, such as fibronectin binding proteins (FnbA, FnbB), serine-aspartate repeat-containing protein D (SdrD), immunoglobulin-binding protein G (Sbi), and other virulence factors. Moreover, several moonlighting proteins, such as translation elongation factors (EF-Tu, EF-G), chaperone protein (DnaK), glyceraldehyde 3-phosphate dehydrogenase (GAPDH), and pyruvate kinase (PK) were detected on the cell surface, while their presence was inversely proportional to surface-associated adhesins. The results obtained from this interesting study suggested that glabridin prevents biofilm formation in *S. aureus* through modulation of the cell surface proteins (Gangwar et al.).

Another study conducted by Yu et al. investigated the inhibitory effect of the novel small-molecule ZY-214-4 (C_19_H_11_BrNO_4_) on a clinical *S. aureus* isolates having biofilm formation ability (confirmed by SEM). This compound significantly suppressed the production of polysaccharides, intercellular adhesion, cell aggregation, as well as inhibited the expression of *icaA* and other biofilm-related genes (*eno, clfA/B, fnbB, fib, ebpS, psm*α, and *psm*β). Authors claim ZY-214-4 as a potent antimicrobial compound highlighting its clinical potential for preventing or treating *S. aureus* infections.

*Staphylococcus epidermidis* is a Gram-positive bacterium frequently associated with biofilm-related infections. The biofilm formation in this bacterium is mediated by the YycG/YycF two-component system, making YycG kinase an attractive therapeutic target. Lyu et al. developed monoclonal antibodies toward to the extracellular domain of the histidine kinase YycG. Both mAbs exhibited dose-dependent inhibition of *S. epidermidis* biofilm formation. The antibodies induced a marked decrease in the synthesis of polysaccharide intercellular adhesin and in the transcriptional level of genes encoding proteins involved in biofilm formation (Lyu et al.).

Dalbavancin (a recently developed lipoglycopeptide antibiotic), is highly effective to prevent staphylococcal (*S. aureus* and *S. epidermidis*) biofilm formation compared to other four antibiotics (linezolid, vancomycin, cloxacillin, and rifampicin). Further experiments were undertaken to check whether biofilm-detaching compounds, such as *N*-acetylcysteine (NAC) and ficin could enhance dalbavancin efficiency. Real-time dose-response experiments showed that dalbavancin is a potent compound preventing staphylococcal biofilm formation. Other exciting results showed that the addition of NAC decreased the efficacy of dalbavancin, while the addition of ficin enhanced its efficacy (Žiemyte et al.). The authors conclude their data support the use of dalbavancin as a favorable antimicrobial compound to treat staphylococcal biofilm infections.

As regarding other Gram-positive bacteria, the work by Qu et al. studied the ability of *Staphylococcus capitis*, an opportunistic pathogen responsible for bloodstream infections in the neonatal intensive care units and able to grow on indwelling central venous catheters, to form biofilms. The authors showed that this bacterium initiated biofilm formation only in response to hyperosmotic conditions. Biofilm development was strongly influenced by the presence of the element oxygen on the surface. On the other hand, a lack of oxidized carbon species on the surface prevented the formation of mature biofilms. Together, this information will be used to suggest guidelines for the preparation of hyperosmolar parenteral nutrition and the engineering of surfaces to minimize the risk of catheter-mediated infections due to *S. capitis*.

## Anti-Pseudomonal and Anti-Biofilm Compounds

Among pathogens able to form biofilm, *Pseudomonas aeruginosa* is one of the most life-threatening, being able to colonize both biotic and abiotic surfaces, including contact lenses, catheters and various medical implants (O'Toole et al., 2000). Interestingly, Su et al. reported that a glycosyl hydrolase produced by this microorganism itself is able to inhibit and disperse biofilms. In particular, they described a mutated version of the hydrolase which is resistant to the action of human secreted proteases, such as trypsin-like serine proteases. Promising effects against both *P. aeruginosa* biofilm formation and virulence factors production were observed by Peppoloni et al. who described a boronic acid derivate designed as a β-lactamase inhibitor. It showed interesting results also against the production of quorum sensing signal molecules, important global regulator of the expression of virulence factors and of biofilm production. Moreover, the achieved data were confirmed also in a model mimicking clinical settings.

It was seen that *P. aeruginosa* was susceptible to the innate immune response molecule nitric oxide (NO). Hassett et al. discussed the NO-based therapeutics. They explain that AB569 can produce NO and can be an alternative or addition to conventional antibiotic regimens to treat highly problematic MDR bacterial infections. The result shows that NO mediate inactivation of the 4Fe-4S cluster of the master regulator, ANR, and NO or its chemical generators have the ability to kill biofilms. RNA sequencing results showed that exposure to AB569 leads to the decrease in transcription of genes involved in the DNA, RNA, proteins, and ATP synthesis. Antimicrobial tolerance of biofilms has emerged as a significant challenge to healthcare sectors as most synthetic drugs and combination therapy fail to inhibit it. Mishra et al. pointed out that natural product-based anti-biofilm agents like phytochemicals, antimicrobial peptides, and microbial enzymes can be an option as they interfere in quorum sensing pathways, disrupting EPS and adhesion mechanisms. It is also seen that most natural product fails in phase II and phase III clinical trials due to the limited availability of the compound in humans. Failure of natural medicine in clinical trials can be checked by rigorous quality control, pharmacokinetics and pharmacodynamics, and metabolomics of host before clinical trials.

## Antibiofilm Compounds from Endophytes

Among natural products, a metabolite produced by the endophytic bacterium *Streptomyces ansochromogenes* has been shown to own both antibacterial and anti-biofilm activity against *P. aeruginosa* (Alves da Fonseca Amorim et al.). Maipomycin A (MaiA) is a novel antibiofilm compound purified from *Kibdelosporangium phytohabitans* XY-R10 through a bio-guided assay. This rare actinomycete strain was isolated from the root sediments of a mangrove plant, *Kandelia candel* (L.) Druce. The authors observed that MaiA has anti-biofilm activities against *Acinetobacter baumannii* and *P. aeruginosa*, and these effects were partially related to its iron chelator property. Although MaiA has weak antimicrobial effects, it is able to enhance the efficacy of colistin against *A. baumannii*. The authors conclude that MaiA is an interesting candidate to prevent Gram-negative biofilms (Zhang et al.). It was seen that pathogens that can present either in planktonic form or as biofilms in water-carrying pipelines are the source accountable for the cause of water-borne infections. Protein-based adhesives from marine mussels, a catecholic amino acid i.e., 3,4-dihydroxyphenylalanine (DOPA) can adhere to almost all substances. A novel catechol derivative, dopamine-based coating material, i.e., polydopamine (PDA), has been designed. Singh et al. discussed the potential of PDA to be used as antibacterial nanocoating and explain its various antimicrobial mechanisms. It was seen that antibacterial activity of PDA is due to the catechol that produces semiquinone and quinone that get auto-oxidized in the presence of oxygen, generation of ROS which causes ROS dependent antibacterial activity (Singh et al.).

## *In vivo* Models and Anti-Biofilm Compounds

Another interesting study is completed by Wang, Gong et al. who investigate the biological impacts of the interactions between two important pathogens (*Streptococcus suis* and *Actinobacillus pleuropneumoniae*) isolated from pigs suffering from severe respiratory disease. More precisely, the authors described that, when grown in dual-species biofilms, *A. pleuropneumoniae* genes associated with virulence factors, including exotoxins and adhesins, were significantly upregulated, while *S. suis* virulence factor-related genes (*cps2, gdh, mrp*, and *sly*) were highly induced. The authors conclude that the interspecies interactions between *S. suis* and *A. pleuropneumoniae* may be achieved under specific conditions and may play a vital role in the disease progression and persistent infection (Wang, Gong et al.).

Aguilera-Correa et al. evaluated the effect of a moxifloxacin-loaded organic-inorganic sol-gel *in vitro* against the formation of different biofilms (*S. aureus, S. epidermidis*, and *Escherichia coli*) and *in vivo* in a murine model. The microbiological studies revealed that sol-gel coatings inhibited the biofilm development and were able to treat mature biofilms of all the three bacterial species. In the *in vivo* study, mice weight increased over time, except in the *E. coli*-infected group without coating. Authors conclude that moxifloxacin-loaded sol-gel coating is capable of preventing both *Staphylococcal* and *E. coli* biofilms in prosthetic joint infection.

## Anti-Fungal and Anti-Biofilm Compounds

The development of chronic and recurrent infections by *Candida albicans* is also attributed to biofilm formation and *C. albicans* is the most prevalent human fungal pathogen in both immunocompetent and immunocompromised individuals (Kerkoub et al., 2018). Despite advances in antifungal therapy, *Candida* infections continue to have a major impact on mortality and morbidity, as well as on the duration and cost of hospitalization (Tanwar et al., 2014). In this special issue, some agents targeting *Candida* biofilm and other virulence determinants were also reported, including RAFT-derived polymethacrylates (Wu et al.), sodium new houttuyfonate (SNH) (Wu et al.), and piperine (Priya and Pandian). In another elegant paper, the activity of Hexyl-aminolevulinate ethosomes system (HAL-ES) as photosensitizer for antimicrobial photodynamic therapy against *C. albicans* (Wang, Song et al.) was reported. The RAFT-Derived Polymethacrylates were effective in two mouse models of Candidiasis: Vulvovaginal candidiasis (VVC) and recurrent VVC (Wu et al.). The SNH showed *in vitro* anti-*Candida* and anti-biofilm activity, inducing morphological changes during the transition from yeast to hypha. These effects are linked to the down-regulation of several biofilm formation related genes from Ras1-cAMP-Efg1 pathway and up-regulation of in yeast form-associated genes (Wu et al.). SNH also has *in vivo* activity in the infection model using *Galleria mellonella*. Similarly, the piperine, a plant-derived alkaloid molecule, displayed antibiofilm activities against *C. albicans* (shown by confocal laser-scanning microscopy and scanning electron microscopy) and modulated the expression of several biofilm related and hyphal-specific genes. In addition, piperine reduced *in vivo* colonization and prolonged the lifespan of *Caenorhabditis elegans* infected by *C. albicans* without any acute toxicity (Priya and Pandian). Transcriptomic analysis revealed that piperine significantly downregulated the expression of several biofilm related and hyphal-specific genes (*ALS3, HWP1, EFG1, CPH1*, etc.).

*Cryptococcus neoformans* is another yeast capable of causing life threatening meningoencephalitis in patients with impaired immunity. In a very elegant study, Villis et al. focused on fungal infections caused by *Cryptococcus* genus. Natural products achieved from the plant *Punica granatum* were investigated for their potential to kill various *Cryptococcus* clinical and environmental isolates and for their activity against pre-formed biofilms, with promising results.

## Anti-Quorum Sensing and Anti-Biofilm Compounds

The ability to inhibit the biofilm of *A. baumannii* was also evaluated for Cec4, an antimicrobial peptide known to have antibacterial and immunomodulatory activities (Liu et al.). Cec4 could inhibit the growth and biofilm formation of a set of 200 carbapenem-resistant *A. baumannii* (CRAB). Importantly, the peptide eradicated the formed biofilm by impairing its structure. Further, Cec4 modulated the expression of 185 genes in CRAB biofilm affecting multiple metabolic pathways, such as two-component regulation systems, quorum sensing, and antibiotic synthesis-related pathways (Liu et al.).

As regarding *E. coli*, another main biofilm-associated opportunistic pathogen, the *Cinnamomum camphora* essential oil was shown to be able to kill clinical strains isolated from dairy cows suffering from endometritis, in either planktonic or biofilm growth conditions, during the first 30 min of exposure (Wang, Zhang et al.). Gram-negative bacterium, *Vibrio harveyi*, has been shown to be subjected to the effect of a natural product, Cannabigerol, which is a non-psychoactive cannabinoid naturally present in the Cannabis plant (Aqawi et al.). Indeed, Aqawi et al. described a reduction in the QS-regulated bioluminescence and biofilm formation of *V. harveyi*. In another work, silver nanoparticles (AgNPs) were produced from leaf extracts of *Semecarpus anacardium, Glochidion lanceolarium*, and *Bridelia retusa* as new approach to eradicate biofilm formed by *P. aeruginosa, E. coli*, and *S. aureus* (Mohanta et al.).

In addition, mixed species biofilms display greater resistance to antibiotics and disinfectants due to physical matrix barrier and physiological interaction. This is one of the major threats to different industries like food and human health. Rao et al. has highlighted the use of cold atmospheric plasma (CAP) to eliminate microbial biofilms by applying it on biotic or abiotic surfaces. They also explain that different microbial factors such as peptidoglycan layer thickness, biofilm thickness, matrix production, etc. affecting the efficacy of the CAP procedure.

To conclude, the present Research Topic includes interdisciplinary research work highlighting the use of several natural derived compounds as future drug candidate. This Research Topic successfully gathered comprehensive interdisciplinary information in the field of drug discovery related to biofilm infections caused by multiple microbial species, such as *S. aureus*, MRSA, *S. capitis, S. epidermidis, S. suis* (as regarding the Gram-positives); *A. baumannii, A. pleuropneumoniae, E. coli, P. aeruginosa, V. harveyi* (for the Gram-negatives). Several studies also include the treatment of fungal biofilm caused by *Candida* and *Cryptococcus* genera. Further, several bioactive compounds were described, as well as *in vivo* studies (*Caenorhabditis elegans, Galleria mellonella* and mice models).

This unique collection of articles in the present Research Topic gives new insights into the characterization of biofilm and drug resistance mechanisms as well as provides novel strategies to fight several notorious pathogens.

We would like to thank all the reviewers for their comments that improved our manuscripts, and the authors for their excellent contributions.

Finally, we hope that this collection will further inspire scientists from different research fields to make use of the gathered information to combat biofilm.

## Author Contributions

All authors listed have made a substantial, direct and intellectual contribution to the work, and approved it for publication.

## Funding

SB was supported by the Italian Ministry of Education, University and Research (MIUR) (Dipartimenti di Eccellenza, Program 2018–2022) L. Spallanzani, University of Pavia.

## Conflict of Interest

The authors declare that the research was conducted in the absence of any commercial or financial relationships that could be construed as a potential conflict of interest.

## Publisher's Note

All claims expressed in this article are solely those of the authors and do not necessarily represent those of their affiliated organizations, or those of the publisher, the editors and the reviewers. Any product that may be evaluated in this article, or claim that may be made by its manufacturer, is not guaranteed or endorsed by the publisher.
